# Global health research abstracts: July ‘23

**DOI:** 10.1016/j.afjem.2023.05.003

**Published:** 2023-06-06

**Authors:** Dr. Jonathan Kajjimu

**Affiliations:** Faculty of Medicine, Mbarara University of Science and Technology, Mbarara, Uganda

## Abstract

The African Journal of Emergency Medicine, in partnership with several other regional emergency medicine journals, publishes abstracts from each respective journal. Abstracts are not necessarily linked to open access papers however, all abstracts are accessible without subscription.

## Annals of emergency medicine (North America)

### Frailty and Neurologic Outcomes of Patients Resuscitated From Nontraumatic Out-of-Hospital Cardiac Arrest: A Prospective Observational Study

Yamamoto, R., Tamura, T., Haiden, A., Yoshizawa, J., Homma, K., Kitamura, N., ... & SOS-KANTO 2017 Study Group. (2023). *Annals of Emergency Medicine*.

DOI: https://doi/10.1016/j.annemergmed.2023.02.009

### Study objective

To elucidate the clinical utility of the Clinical Frailty Scale score for predicting poor neurologic functions in patients resuscitated from out-of-hospital cardiac arrest (OHCA).

### Methods

This was a prospective, multicenter, observational study conducted between 2019 and 2021. The study included adults with nontraumatic OHCA admitted to the intensive care unit after return of spontaneous circulation (ROSC). Pre-arrest high Clinical Frailty Scale score was defined as 5 or more. Favorable neurologic outcomes defined as a Cerebral Performance Category score of 2 or less at 30 days after admission were compared between patients with and without high Clinical Frailty Scale scores. Multivariable logistic regression analyses fitted with generalized estimating equations were performed to adjust for patient characteristics, out-of-hospital information, and resuscitation content and account for within-institution clustering.

### Results

Of 9,909 patients with OHCA during the study period, 1,216 were included, and 317 had a pre-arrest high Clinical Frailty Scale score. Favorable neurologic outcomes were fewer among patients with high Clinical Frailty Scale scores. The high Clinical Frailty Scale score group showed a lower percentage of favorable neurologic outcomes after OHCA than the low Clinical Frailty Scale score group (6.1% vs 24.4%; adjusted odds ratio, 0.45 [95% confidence interval 0.22 to 0.93]). This relationship remained in subgroups with cardiogenic OHCA, with ROSC after hospital arrival, and without a high risk of dying (Clinical Frailty Scale score of 7 or less), whereas the neurologic outcomes were comparable regardless of pre-arrest frailty in those with noncardiogenic OHCA and with ROSC before hospital arrival.

### Conclusions

Pre-arrest high Clinical Frailty Scale score was associated with unfavorable neurologic functions among patients resuscitated from OHCA. The Clinical Frailty Scale score would help predict clinical consequences following intensive care after ROSC.

Reproduced with permission.

## Canadian Journal of Emergency Medicine (North America)

A value-based comparison of the management of respiratory diseases in walk-in clinics and emergency departments.

Marx, T., Moore, L., Talbot, D., Guertin, J. R., Lachapelle, P., Blais, S., ... & Berthelot, S. (2023). *Canadian Journal of Emergency Medicine*, 1-9.

DOI: https://doi.org/10.1007/s43678-023-00481-7

### Objectives

Our aim was to compare some of the health outcomes and costs associated with value of care in emergency departments (ED) and walk-in clinics for ambulatory patients presenting with an acute respiratory disease.

### Methods

A health records review was conducted from April 2016 through March 2017 in one ED and one walk-in clinic. Inclusion criteria were: (i) ambulatory patients at least 18 years old, (ii) discharged home with a diagnosis of upper respiratory tract infection (URTI), pneumonia, acute asthma, or acute exacerbation of chronic obstructive pulmonary disease. Primary outcome was the proportion of patients returning to any ED or walk-in clinic within three and seven days of the index visit. Secondary outcomes were the mean cost of care and the incidence of antibiotic prescription for URTI patients. The cost of care was estimated from the Ministry of Health's perspectives using time-driven activity-based costing.

### Results

The ED group included 170 patients and the walk-in clinic group 326 patients. The return visit incidences at three and seven days were, respectively, 25.9% and 38.2% in the ED vs. 4.9% and 14.7% in the walk-in clinic (adjusted relative risk (arr) of 4.7 (95% CI 2.6–8.6) and 2.7 (1.9–3.9)). The mean cost ($Cdn) of the index visit care was 116.0 (106.3–125.7) in the ED vs. 62.5 (57.7–67.3) in the walk-in clinic (mean difference of 56.4 (45.7–67.1)). Antibiotic prescription for URTI was 5.6% in the ED vs. 24.7% in the walk-in clinic (arr 0.2, 0.01–0.6).

### Conclusions

This study is the first in a larger research program to compare the value of care between walk-in clinics and the ED. The potential advantages of walk-in clinics over EDs (lower costs, lower incidence of return visits) for ambulatory patients with respiratory diseases should be considered in healthcare planning.

Reproduced with permission.

## Emergencias (Europe)

Safety and efficiency of discharge to home hospitalization of patients with acute heart failure directly after emergency department care.

Sánchez Marcos C, Espinosa B, Coloma E, San Inocencio D, Pilarcikova S, Guzmán Martínez S, et al. Emergencias. DOI: 891.

https://bit.ly/3AOfS0x

### Objectives

To analyze whether discharge to home hospitalization (HHosp) directly from emergency departments (EDs) after care for acute heart failure (AHF) is efficient and if there are short-term differences in outcomes between patients in HHosp versus those admitted to a conventional hospital ward (CHosp).

### Methods

Secondary analysis of cases from the EAHFE registry (Epidemiology of Acute Heart Failure in Emergency Departments). The EAHFE is a multicenter, multipurpose, analytical, non-interventionist registry of consecutive AHF patients after treatment in EDs. Cases were included retrospectively and registered to facilitate prospective follow-up. Included were all patients diagnosed with AHF and discharged to HHosp from 2 EDs between March 2016 and February 2019 (3 years). Cases from 6 months were analyzed in 3 periods: March-April 2016 (corresponding to EAHFE-5), January-February 2018 (EAHFE-6), and January-February 2019 (EAHFE-7). The findings were adjusted for characteristics at baseline and during the AHF decompensation episode.

### Results

A total of 370 patients were discharged to HHosp and 646 to CHosp. Patients in the HHosp group were older and had more comorbidities and worse baseline functional status. However, the decompensation episode was less severe, triggered more often by anemia and less often by a hypertensive crisis or acute coronary syndrome. The HHosp patients were in care longer (median [interquartile range], 9 [7-14] days vs 7 [5-11] days for CHosp patients, P < .001), but there were no differences in mortality during hospital care (7.0% vs. 8.0%, P = .56), 30-day adverse events after discharge from the ED (30.9% vs. 32.9%, P = .31), or 1-year mortality (41.6% vs. 41.4%, P = .84). Risks associated with HHosp care did not differ from those of CHosp. The odds ratios (ORs) were as follows: mortality while in hospital care, OR 0.90 (95% CI, 0.41-1.97); adverse events within 30 days of ED discharge, OR 0.88 (95% CI,

0.62-1.26); and 1-year mortality, OR 1.03 (95% CI, 0.76-1.39). Direct costs of HHosp and CHosp averaged €1309 and €5433, respectively.

### Conclusion

After ED treatment of AHF, discharge to HHosp requires longer care than CHosp, but short- and long term outcomes are the same and at a lower cost.

Reproduced with permission.

## Emergency Medicine Journal (Europe)

DOI: http://dx.doi.org/10.1136/emermed-2022-212718

### Background

Disparate care in the ED for minority populations with low back pain is a long-standing issue reported in the USA. Our objective was to compare care delivery for low back pain in Australian EDs between culturally and linguistically diverse (CALD) and non-CALD patients.

### Methods

This is a retrospective review of medical records of the ED of three public hospitals in Sydney, New South Wales, Australia from January 2016 to October 2021. We included adult patients diagnosed with non-serious low back pain at ED discharge. CALD status was defined by country of birth, preferred language and use of interpreter service. The main outcome measures were ambulance transport, lumbar imaging, opioid administration and hospital admission.

### Results

Of the 14, 642 included presentations, 7,656 patients (52.7%) were born overseas, 3,695 (25.2%) preferred communicating in a non-English language and 1,224 (8.4%) required an interpreter. Patients born overseas were less likely to arrive by ambulance (adjusted OR (aOR) 0.68, 95% CI 0.63 to 0.73) than Australian-born patients. Patients who preferred a non-English language were also less likely to arrive by ambulance (aOR 0.82, 95% CI 0.75 to 0.90), yet more likely to be imaged (aOR 1.12, 95% CI 1.01 to 1.23) or be admitted to hospital (aOR 1.16, 95% CI 1.04 to 1.29) than Native-English-speaking patients. Patients who required an interpreter were more likely to receive imaging (aOR 1.43, 95% CI 1.25 to 1.64) or be admitted (aOR 1.49, 95% CI 1.29 to 1.73) compared with those who communicated independently. CALD patients were generally less likely to receive weak opioids than non-CALD patients (aOR range 0.76–0.87), yet no difference was found in the use of any opioid or strong opioids.

### Conclusion

Patients with low back pain from a CALD background, especially those lacking English proficiency, are significantly more likely to be imaged and admitted in Australian EDs. Future interventions improving the quality of ED care for low back pain should give special consideration to CALD patients.

Reproduced with permission.

## Hong Kong journal of emergency medicine (Asia)

Positive impact of trauma center to exsanguinating pelvic bone fracture patient survival: A Korean trauma center study.

Lee, M., Yu, B., Lee, G., Lee, J., Choi, K., Park, Y., ... & Jang, M. J. (2022). *Hong Kong Journal of Emergency Medicine*, 10249079221087799.

DOI: https://doi.org/10.1177/10249079221087799

### Background

Trauma center and multidisciplinary management protocols have been proven to improve the outcomes of severely injured patients. Hemorrhage from pelvic injury is associated with high mortality and is a common cause of preventable trauma death. This study aimed to evaluate the effects of the establishment of a trauma center and management protocols on the outcomes of hemodynamically unstable patients with pelvic fractures.

### Methods

Hemodynamically unstable patients with pelvic fractures were reviewed retrospectively over a 10-year period. They were grouped into the pre-phase and post-phase, which were defined as before and after the establishment of a trauma center and protocols, respectively. Basic characteristics and outcomes were compared between periods.

### Results

This study enrolled a total of 106 patients. Basic and physiological characteristics were not significantly different in both phases. Pre-peritoneal packing and resuscitative endovascular balloon occlusion of aorta were only performed in the post-phase (pre-peritoneal packing, N = 27; resuscitative endovascular balloon occlusion of aorta, N = 10). In the post-phase, the time from emergency department arrival to hemostatic intervention was significantly shorter (269 ± 132.4 min vs 147.2 ± 95.5 min, p < 0.0001), and mortality due to acute hemorrhage was significantly lower (p = 0.003; absolute risk reduction: 0.22; relative risk reduction: 0.72). Multivariate logistic regression analysis identified age, injury severity score, and the pre-phase as independent risk factors for mortality.

### Conclusion

The establishment of a trauma center and multidisciplinary management protocols, such as pre-peritoneal packing and resuscitative endovascular balloon occlusion of aorta, improved the outcomes of hemodynamically unstable patients with pelvic fractures.

Reproduced with permission.

## African relevance

Not applicable.

## Dissemination of results

Not applicable.

## Declaration of Competing Interest

Not applicable

